# Assessment of Bicarbonate Deficiency in Feline Acute and Chronic Kidney Disease

**DOI:** 10.3390/vetsci12111097

**Published:** 2025-11-18

**Authors:** Francesca Perondi, Matilde Vernaccini, Silvia Morelli, Veronica Marchetti, Ilaria Lippi

**Affiliations:** Department of Veterinary Sciences, University of Pisa, San Piero a Grado, 56122 Pisa, Italy; f.perondi87@gmail.com (F.P.); silviamorelli.vet@gmail.com (S.M.); veronica.marchetti@unipi.it (V.M.); ilaria.lippi@unipi.it (I.L.)

**Keywords:** bicarbonate, cat, AKI, CKD, metabolic acidosis, calcium-phosphate product

## Abstract

Bicarbonate deficiency is a common complication of acute kidney injury (AKI) and chronic kidney disease (CKD), resulting from impaired renal bicarbonate handling. It promotes bone demineralization and calcium–phosphate (CaxP) imbalance. In dogs, elevated CaxP has been linked to increased frequency and severity of bicarbonate deficiency. This 10-year study evaluated the prevalence and degree of bicarbonate deficiency in 618 cats diagnosed with AKI, acute-on-chronic kidney disease (ACKD), and CKD, and its association with azotemia and mineral imbalance. A retrospective analysis was conducted on serum biochemical profiles, including creatinine, urea, ionized and total calcium, phosphate, CaxP, and bicarbonate. Cats with incomplete datasets or receiving sodium bicarbonate therapy were excluded. According to IRIS guidelines, bicarbonate deficiency was defined as <16 mmol/L and categorized as moderate (12–16 mmol/L) or severe (<12 mmol/L). Deficiency was found in 45% of cats, of which 63% were moderate and 37% severe. Prevalence was higher in AKI (58%) and ACKD (60%) compared to CKD (38%) (*p* = 0.002). Negative correlations between bicarbonate and creatinine, phosphate, and CaxP were detected in AKI and CKD, while in ACKD, only creatinine and phosphate correlated. Deficiency increased with disease severity in AKI (*p* = 0.0145) and CKD (*p* < 0.0001). Cats with CaxP > 70 mg^2^/dL^2^ had higher deficiency rates (*p* < 0.0001). However, 21% with elevated CaxP had normal bicarbonate, suggesting mineral imbalance may occur independently of acidosis.

## Simple Summary

The kidneys are essential for regulating acid–base homeostasis, and their impairment may result in reduced bicarbonate levels and metabolic acidosis. This condition is well known in humans and dogs and is associated with bone and mineral disorders. Our study evaluated cats with acute and chronic kidney disease to understand how often bicarbonate deficiency occurs and whether it is linked to mineral imbalances. We found that almost half of the cats were affected, with higher prevalence in acute conditions. Altered calcium–phosphate homeostasis was associated with a higher occurrence of bicarbonate deficiency, indicating a relationship between acidosis and bone–mineral imbalance in cats.

## 1. Introduction

Physiologically, the kidneys maintain acid–base equilibrium by controlling acid elimination and bicarbonate recycling and synthesis within the renal tubules. Beyond their role in acid–base balance, the kidneys contribute significantly to mineral and bone metabolism through phosphate excretion and vitamin D activation. As functional nephron mass is progressively lost in patients with chronic kidney disease (CKD), this often results in impaired acid–base regulation and the subsequent development of metabolic acidosis [1]. Although a comprehensive diagnosis of metabolic acidosis requires both serum bicarbonate measurement and blood pH evaluation, the lack of access to blood gas analyses in routine clinical settings often limits the assessment. Consequently, serum bicarbonate, typically included in standard biochemistry panels for renal evaluation, is commonly used as a surrogate marker of acid–base status. Metabolic acidosis, usually defined by a serum bicarbonate concentration below 22 mmol/L, is a frequent complication of CKD in both humans and animals, with its occurrence rising in the later stages of the disease [1,2]. This acid–base imbalance is associated with a range of systemic complications. In both human and veterinary medicine, metabolic acidosis has been linked to enhanced protein catabolism, chronic inflammation, anemia of chronic disease, and disturbances in bone metabolism, including bone demineralization and mineral–bone disorders [2,3,4]. While these effects are well documented in human medicine, their pathophysiological implications in veterinary patients remain an active area of investigation. Several studies have demonstrated that disturbances in acid–base homeostasis are associated with a faster progression of CKD and increased mortality among affected patients [1,5]. In feline medicine, according to the International Renal Interest Society (IRIS) guidelines [6], serum bicarbonate concentrations below 16 mmol/L are considered indicative of metabolic acidosis.

Given the high prevalence of metabolic acidosis and mineral metabolism disorders reported in both human and canine patients with renal disease [7], the present study aims to evaluate the prevalence of metabolic acidosis in cats diagnosed with acute kidney injury (AKI), acute-on-chronic kidney disease (ACKD), and chronic kidney disease (CKD). Furthermore, it seeks to explore potential associations between metabolic acidosis, IRIS stage or grade of renal dysfunction, and concurrent mineral metabolism abnormalities.

## 2. Materials and Methods

In this retrospective study, medical records of all feline patients presented to the “Mario Modenato” Veterinary Teaching Hospital (VTH) at the University of Pisa between August 2013 and November 2023 were systematically reviewed. All data were collected through the database management software of the VTH (OCIROE). Cats were eligible for inclusion if they had a diagnosis of acute kidney injury (AKI), acute-on-chronic kidney disease (ACKD), or chronic kidney disease (CKD), and if a complete serum biochemical profile was available, including at minimum: creatinine (mg/dL), urea (mg/dL), total calcium (mg/dL), phosphate (mg/dL), and bicarbonate (TCO_2_, mmol/L). When available, symmetric dimethylarginine (SDMA) (mcg/dL) and calcium-phosphate product (CaxP) (mg^2^/dL^2^) were also recorded. For all included patients (*n* = 618), additional data such as age, body weight, and diagnosis were extracted from clinical records.

Cats were assigned to the AKI, ACKD, or CKD groups according to clinical history, laboratory data, and imaging results. Only cats that had undergone abdominal ultrasonography within 15 days of blood sample collection were considered for inclusion. Biochemical panels were excluded if any of the required parameters were missing or if the sample was collected after a dialysis session. Blood samples were collected from the jugular, cephalic, or saphenous vein and immediately transferred into 2.5 mL methacrylate tubes. Samples were centrifuged within 15 min of collection and analyzed using the SAT 450 automatic biochemical analyzer (Assel, Rome, Italy).

In cases of AKI or ACKD, the underlying etiology of renal dysfunction was recorded when known. Cats were assigned to the AKI group if they met the following criteria: (1) a sudden onset of clinical signs consistent with AKI (e.g., vomiting, lethargy, oliguria, or anuria); (2) absence of ultrasonographic findings typically associated with chronic kidney disease (e.g., ultrasound abnormalities included increased cortical echogenicity, diminished cortico-medullary definition, decreased or asymmetric renal size, and renal cyst formation) [8]; and (3) presence of azotemia, defined as a serum creatinine concentration exceeding 1.7 mg/dL. Cats showing ultrasonographic evidence of CKD but with a documented episode of acute clinical deterioration within the previous three months were categorized as having ACKD. Cats without clinical signs suggestive of acute deterioration and presenting only with features consistent with chronic renal dysfunction were assigned to the CKD group. Patients in the AKI and ACKD groups were stratified according to the IRIS grading system [6] into grades 1 through 5 based on serum creatinine concentrations. Conversely, cats in the CKD group were classified into IRIS stages 1 through 4 [9].

Metabolic acidosis severity was defined based on serum bicarbonate levels: moderate acidosis was considered present when bicarbonate ranged from 12 to 16 mmol/L, and severe acidosis when levels were below 12 mmol/L. A CaxP value equal to or greater than 70 mg^2^/dL^2^ was considered indicative of mineral metabolism disturbance [10].

The distribution of continuous variables was assessed using the Kolmogorov–Smirnov test for normality. As none of the variables followed a normal distribution, data were reported as medians along with minimum and maximum values. Comparisons of median values among the three groups (AKI, ACKD, and CKD) were conducted using the Kruskal–Wallis test. Fisher’s exact test was employed to evaluate differences in the frequency and severity of serum bicarbonate deficiency among the groups, as well as between cats with normal and abnormal calcium-phosphate product (CaxP) values. Correlations between serum bicarbonate and creatinine, total calcium, phosphate, and CaxP within each group were analyzed using Spearman’s correlation test. Statistical analyses were performed using GraphPad Prism 7^®^ software, with significance set at a *p*-value < 0.05.

## 3. Results

In the present study, a total of 1.787 clinical records were reviewed. Of these, 1.046 were excluded due to incomplete data, 120 were excluded either because imaging records were unavailable or because they presented only a mild increase in serum creatinine (1.71–1.99 mg/dL) without ultrasonographic evidence of renal pathology, and 3 were excluded as they referred to post-dialysis assessments. Ultimately, 618 clinical records met the inclusion criteria and were retained for analysis. The clinical records were categorized into AKI (*n* = 83), ACKD (*n* = 116) and CKD (*n* = 419). All relevant data about gender, weight and age of the cats included in the different study groups are summarized in Table 1.

In the AKI group the renal failure was due to not known causes in 28/83 cats (34%), to urethral or ureteral obstruction in 27/83 cats (33%), to inflammatory/infective disease in 21/83 cats (25%) and cancer in 3/83 (4%), single cats also had renal failure due to intestinal foreign objects, intoxication, bladder rupture or trauma. In the ACKD group the causes of renal failure were distributed as follows: inflammatory/infective disease in 58/116 cats (50%), not known in 26/116 cats (23%), urethral or ureteral obstruction/sub-obstruction in 15/116 (13%), a combination of inflammation and obstruction in 9/116 cats (8%), FIV in 4/116 (3%) and other causes in 4/116 cats (3%).

Serum bicarbonate < 16 mmol/L was found in 276/618 cats (45%): 173/276 cats (63%) showed a moderate deficiency, while 103/276 cats (37%) had a severe deficiency. Serum bicarbonate deficiency was found in 48/83 cats (58%) of the AKI group, in 32 of them (67%) the deficiency was moderate, while in 16 of them (33%) it was severe. In the ACKD group the deficiency of serum bicarbonate was found in 70/116 cats (60%), 37/70 cats (53%) showed a moderate deficiency, while 33/70 cats (47%) had a severe deficiency. In the CKD group, serum bicarbonate deficiency was found in 158/419 cats (38%), with moderate deficiency in 104/158 cats (66%) and severe deficiency in 54/158 cats (34%). Adhering to the IRIS classification criteria [6], cats exhibiting reduced serum bicarbonate levels were categorized accordingly. The AKI cohort comprised *n* = 12 Grade 2 cases, *n* = 7 Grade 3, *n* = 13 Grade 4, and *n* = 16 Grade 5. ACKD patients demonstrated a distribution across Grade 2 (*n* = 7), Grade 3 (*n* = 12), Grade 4 (*n* = 27), and Grade 5 (*n* = 24). Conversely, the CKD group was distributed across Stage 2 (*n* = 79), Stage 3 (*n* = 31), and Stage 4 (*n* = 48).

Table 2, Table 3 and Table 4 show the clinical data of all patients included in the study, categorized by group (AKI, CKD, and ACKD) and compared based on the presence and severity of metabolic acidosis, the values of serum creatinine, urea, CaxP and TCO_2_ in the patients included in the study, grouped according to diagnosis (AKI, CKD, and ACKD), and the correlation between serum bicarbonate and creatinine, CaxP, total calcium, and phosphate among the three groups, respectively.

**Figure 1:**
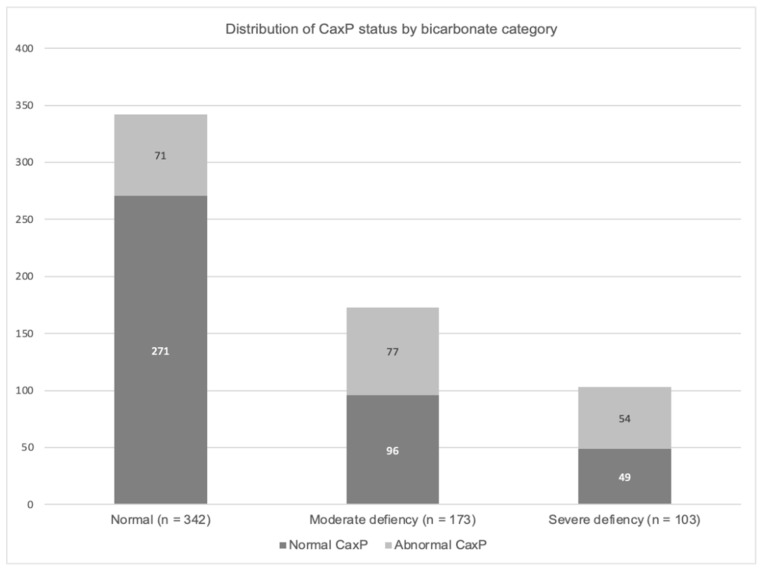
Prevalence of serum CaxP abnormality in patients with different levels of serum bicarbonate deficiency.

CaxP values were available for all 618 cats included in the study. Among the 342 cats with normal serum bicarbonate, 71 (21%) showed an abnormal CaxP (≥70 mg^2^/dL^2^), while the remaining 271 cats (79%) had normal CaxP levels. In contrast, among the 276 cats with reduced serum bicarbonate, 131 (47%) exhibited abnormal CaxP values. Of the 131 cats with both bicarbonate deficiency and abnormal CaxP, 77 (59%) had moderate bicarbonate deficiency (12–16 mmol/L), and 54 (41%) had severe deficiency (<12 mmol/L). Among cats with moderate bicarbonate deficiency (*n* = 173), 96 (55%) had normal CaxP levels, whereas 77 (45%) had elevated CaxP. In the subgroup with severe bicarbonate deficiency (*n* = 103), 49 cats (48%) had normal CaxP values, while 54 (52%) showed abnormal CaxP levels. Figure 1 illustrates the distribution of CaxP status, classified as normal or abnormal (≥70 mg^2^/dL^2^), across three serum bicarbonate categories. The graph shows a rising trend of abnormal CaxP values with increasing bicarbonate deficiency, suggesting a link between metabolic acidosis and mineral metabolism imbalance in cats with renal disease. Cats with a serum Ca × P product ≥ 70 mg^2^/dL^2^ showed a significantly higher prevalence of metabolic acidosis compared to those with Ca × P < 70 mg^2^/dL^2^ (*p* < 0.0001) (Table 5). However, the severity of acidosis did not differ significantly between the two groups, with moderate acidosis remaining more frequent than severe acidosis in both categories (*p* = 0.2147).

## 4. Discussion

Bicarbonate deficiency is a frequent consequence of renal disease, as impaired kidney function disrupts acid–base regulation. Metabolic acidosis is strongly linked to renal dysfunction. Moderate forms predominate even in advanced stages, suggesting that disease etiology and comorbidities influence severity.

In our study population, cats in the AKI and CKD groups were predominantly classified in the early stages of renal disease (IRIS grade 2–3 and stage 2, respectively), whereas those in the ACKD group were more frequently classified in advanced stages (grade 4–5). The high proportion of cats with early-stage AKI was unexpected, given that acute kidney injury is typically associated with rapid and severe deterioration of renal function. This finding may be attributed to the timing of biochemical profiling, which in many cases occurred several hours after patient admission, likely due to logistical aspects of the VTH. Regarding the CKD group, the relatively low number of cats with advanced-stage disease could be explained by follow-up loss or by the slow progression of CKD in feline patients, particularly when diagnosed early and appropriately managed [11]. In contrast, the predominance of advanced IRIS grades among cats with ACKD likely reflects the presence of pre-existing CKD. In such cases, the superimposed acute event leads to a marked increase in serum creatinine from already elevated baseline values.

The overall prevalence of bicarbonate deficiency in cats with renal disease was 45%, which appears lower than the prevalence reported in dogs (76%) [7]. In human medicine, the prevalence of metabolic acidosis among CKD patients varies widely, ranging from 2.3% to 46.6%, depending on the population studied and the stage of disease [12,13]. However, these differences should be interpreted with caution, as the definition of metabolic acidosis differs among species. In both dogs and humans, metabolic acidosis is typically defined by a serum TCO_2_ level below 22 mmol/L. In contrast, this study employed a lower cutoff of <16 mmol/L for cats, as recommended by the most recent IRIS guidelines [6]. The use of this more conservative threshold may have contributed to an underestimation of the true prevalence of metabolic acidosis in feline patients compared to dogs and humans. The lower prevalence of metabolic acidosis observed in cats compared to dogs may be influenced by differences in population distribution. In Lippi et al. (2023) [7], the canine population included 19% with AKI, 28% with ACKD, and 53% with CKD, whereas in the present feline study, 13% had AKI, 19% ACKD, and 68% CKD. Given that CKD is associated with the lowest prevalence of acidosis in both studies, its higher representation in the feline cohort may have contributed to the overall lower prevalence of metabolic acidosis. The high prevalence of bicarbonate deficiency observed in this study is consistent with existing evidence in both human and veterinary medicine, where a strong association between metabolic acidosis and renal failure is well-documented. This link reflects the kidney’s central role in acid–base regulation through acid excretion, bicarbonate reabsorption, and bicarbonate synthesis from glutamine [13].

The median TCO_2_ and CaxP values in the CKD group were significantly different from those in the AKI and ACKD groups, and only in the CKD group did these medians fall within the reference range. This may reflect the more stable condition of CKD cats, who have likely activated compensatory mechanisms over time, unlike AKI and ACKD patients, who may be in the acute phase of disease. In AKI and ACKD cats, bicarbonate deficiency may also result from non-renal causes, such as diarrhea or hyperlactataemia. The predominance of early-stage disease in the CKD group, along with dietary management (e.g., renal diets), may explain the more balanced acid–base and mineral profiles. Although cats in the ACKD group may also receive dietary support, it is likely insufficient to offset the effects of an acute insult. Moreover, in ACKD, mineral–bone disorders may have been subclinical before the acute event, as FGF-23 (Fibroblast Growth Factor-23) typically rises before CaxP abnormalities become evident [14]. CKD cats may also have early-stage mineral imbalances that are not yet detectable through CaxP levels.

Our study identified a negative linear correlation between TCO_2_ and creatinine across all groups, with the strongest correlation in AKI and the weakest in CKD. This suggests that while declining renal function contributes to bicarbonate deficiency, additional factors are involved in modulating acid–base balance. In dogs, such factors include hyperlactatemia, gastrointestinal bicarbonate loss, hyperphosphatemia, and acute pancreatitis [7,15]. A similar trend was observed in the correlation between TCO_2_ and phosphorus, reflecting their shared dependence on glomerular filtration. However, CaxP may offer a more comprehensive indication of mineral metabolism disturbances than phosphorus alone [16]. A significant negative correlation between TCO_2_ and CaxP was observed in both the AKI and CKD groups (*p* = 0.0074 and *p* = 0.0007), indicating an association between metabolic acidosis and mineral metabolism disturbances. This is further supported by Fisher’s test, which showed that cats with CaxP ≥ 70 mg^2^/dL^2^ are more likely to develop acidosis than those with normal values. These findings align with previous studies linking renal dysfunction, acidosis, and mineral–bone disorders, where bone acts as a buffer by releasing calcium and phosphate in response to acidosis [7,17]. Additionally, reduced GFR in renal patients contributes to phosphorus retention may exacerbate bone demineralization. 21% of cats without bicarbonate deficiency had a CaxP product ≥70 mg^2^/dL^2^, suggesting that mineral–bone disorders can occur independently of overt acidosis. This finding supports the inclusion of CaxP assessment in the routine evaluation of early-stage renal patients and warrants further investigation into its relationship with acid–base status.

Hyperphosphatemia and hypocalcemia, resulting from impaired renal activation of vitamin D, trigger increased production of FGF-23 and parathyroid hormone (PTH), leading to renal secondary hyperparathyroidism (RSHP) [18]. Initially compensatory, this mechanism ultimately reduces bone density and elevates serum phosphorus levels. Persistently elevated CaxP product (>70 mg^2^/dL^2^) is linked to calcium phosphate deposition in vessels, joints, soft tissues, and renal parenchyma, worsening CKD prognosis in both humans and animals [10,19]. The lack of correlation between reduced TCO_2_ and increased CaxP in the ACKD group likely reflects its heterogeneity, combining cats with stable chronic disease and those with acute episodes. Although no significant association was found between acidosis severity and CaxP abnormalities, there is a trend toward more severe acidosis in cats with mineral–bone disorders, consistent with canine data [7].

The median body weights differed significantly among the AKI, ACKD, and CKD groups. Cats in the CKD group had the lowest median weight (3.9 kg), whereas those in the AKI group had the highest (4.55 kg). The lower median weight observed in the CKD group aligns with previous findings in cats [19,20], dogs [21], and humans [22]. In chronic disease, progressive weight loss is primarily attributed to increased protein catabolism driven by chronic inflammation [16,23]. In humans, metabolic acidosis has also been shown to promote protein catabolism [4]. In dogs, weight loss associated with chronic disease correlates with reduced life expectancy [24]. As expected, the AKI group showed the highest median weight, likely reflecting the acute nature of the renal insult and the limited time for significant catabolic effects to occur before clinical presentation.

The median ages of the three groups differed significantly, with the CKD group presenting the highest median age (12 years). This finding aligns with data from the Centres for Disease Control and Prevention’s CKD Surveillance System (2021) [25], confirming the well-established association between ageing and chronic diseases, including CKD, in both humans and animals. Age-related physiological changes, such as increased production of reactive oxygen species (ROS), contribute to this relationship [26]. ROS overproduction is associated with reduced expression of klotho, a protective factor against CKD progression [27], and the development of chronic inflammation, a driver of renal fibrosis [28]. The older age observed in CKD patients may also reflect the cumulative effect of multiple unresolved acute kidney injuries over time, eventually leading to overt chronic disease [29]. Some cats initially classified with AKI may later be reclassified as CKD if renal function fails to recover, an outcome reported in 41.2% of human AKI patients [30]. Consistent with findings in humans from developing countries, the youngest median age was found in the AKI group of our study [30]. This may be due to younger individuals’ increased exposure to nephrotoxic or infectious agents. In cats specifically, the higher frequency of urethral obstruction in younger animals may also contribute. In veterinary medicine, the underlying cause of AKI often remains undetermined due to diagnostic and financial limitations. Published studies indicate that the most common causes of feline AKI include toxic substance (e.g., ethylene glycol) and toxins ingestion (e.g., lilies, grapes), ischemic events, infections such as pyelonephritis, and lower urinary tract obstruction [31]. In our study, these findings were confirmed: 34% of AKI cases had no identifiable cause, 33% were due to obstruction, 20% were classified as infectious/inflammatory, and 13% had other causes.

Our study demonstrated a positive correlation between the severity of renal dysfunction and the prevalence of metabolic acidosis in both the AKI and CKD groups. This association was not evident in the ACKD group, likely due to its heterogeneity, as it includes cats at various CKD stages experiencing acute events of differing severity. In the CKD group, the significant difference in acidosis prevalence across stages (*p* < 0.0001) may be attributed to the high proportion of stage 2 cats (67%) without acidosis. In the AKI group, the high rate of acidosis in grade 5 cats (76%) strongly influenced the overall trend, aligning with findings in dogs [7] and in human studies [32]. These results reinforce the concept that declining renal function is closely linked to impaired acid–base regulation. No statistically significant association was found between acidosis severity and IRIS stage or grade in any group. In both the AKI and CKD groups, moderate acidosis was more prevalent than severe acidosis, even in the most advanced stages. Although ACKD cats in IRIS stage 5 more frequently exhibited severe acidosis (39%) compared to moderate (28%), this difference was not statistically significant. Moderate acidosis was more common across all groups, consistent with findings in humans, where most CKD patients exhibit moderate acidosis even in later stages [1]. This contrasts with canine data, where severe acidosis is more prevalent [7]. Species differences in the underlying causes of AKI, such as the predominance of urinary obstruction in cats versus ischemic or inflammatory causes in dogs [33], and differing comorbidities may account for these discrepancies. Contrary to expectations, the prevalence of severe acidosis was not higher in the AKI group compared to the ACKD group (19% vs. 28%). This may reflect differing clinical backgrounds: AKI cats likely had previously healthy kidneys, whereas ACKD cats had pre-existing renal impairment. Our findings suggest that kidneys with preserved baseline function may better buffer acute bicarbonate loss, while chronically compromised kidneys may be less able to maintain acid–base homeostasis following acute insults.

In our study, bicarbonate deficiency was common even in the early stages of renal failure. This aligns with findings in dogs by Lippi et al. (2023) [7] and suggests factors beyond reduced GFR contribute to acid–base imbalance. In human medicine, a high dietary acid load has been implicated in acidosis development in patients with compromised acid–base regulation [1], a factor worth investigating in cats not on renal diets. Additionally, early acute renal failure may involve extrarenal causes of acidosis such as bicarbonate loss via diarrhea [16], lactic acidosis from tissue hypoperfusion [34], and cellular H^+^/K^+^ exchange in response to hyperkalemia. These findings underscore the importance of assessing acid–base status even in mild renal disease to avoid overlooking a potential progression factor [35].

### Study Limitations

This study is subject to several limitations. First, the unequal group sizes (AKI, ACKD, and CKD) may have influenced the overall assessment of acidosis prevalence and severity distribution. Second, the underlying cause of renal failure remained unidentified in many AKI and ACKD cases, which prevented a comprehensive analysis of its impact on the development of acidosis. Furthermore, the lack of detailed comorbidity data restricts the interpretation of their potential role in the acid–base balance of the patients. Complete blood gas analysis was not available for every patient. Consequently, the study utilized serum bicarbonate concentration as the primary indicator of metabolic acidosis. Finally, due to hospital workflow, some biochemical profiles were collected 12 to 48 h after the initiation of medical treatment (such as fluid therapy, bicarbonate administration), which introduces a potential bias. This pre-analytical treatment could have led to an underestimation of the true severity of the initial acidosis observed in the cats.

## 5. Conclusions

Serum bicarbonate deficiency is frequently observed in cats diagnosed with both acute and chronic kidney disease, which aligns with findings reported in both human and canine medicine. The reduction in serum bicarbonate appears to be primarily related to the rate of functional renal mass loss, explaining its higher prevalence in acute conditions. As hypothesized, a CaxP ≥ 70 mg^2^/dL^2^ was associated with an increased frequency of bicarbonate deficiency. Notably, a substantial proportion of cats with normal bicarbonate levels also exhibited elevated CaxP, suggesting that mineral–bone disorders may develop earlier than typically recognized. These findings highlight the potential value of monitoring and managing acid–base balance to mitigate disturbances in mineral metabolism. Longitudinal studies are warranted to explore whether improved control of metabolic acidosis translates into better clinical outcomes in feline renal patients.

## Figures and Tables

**Table 1:** Summary of demographic data for cats included in the study, categorized by diagnostic group (AKI, ACKD, CKD). The table reports the number of cats by sex (intact or neutered) and the median (range) of age (years) and body weight (kg) for each group. The *p*-value denotes statistical significance in the Kruskal–Wallis test, whereas superscript letters indicate significant differences identified by Dunn’s multiple comparison test.

Group	Gender		Weight (kg)	Age (Years)
	Males (*n* = 363)	Females (*n* = 255)		
	Intact/Neutered	Intact/Neutered		
AKI (*n* = 83)	15/44	1/23	4.55 ^a^ (2.30–8.00)	4.8 ^a^ (0–18)
ACKD (*n* = 116)	16/51	4/45	4.26 ^a^ (2.29–9.30)	10.3 ^b^ (0–18)
CKD (*n* = 419)	44/193	15/167	3.90 ^b^ (1.55–10.98)	12.7 ^c^ (0–21)
*p*-value			0.040	<0.0001

**Table 2:** Distribution of the cats in the 3 groups (AKI, ACKD, CKD) according to IRIS classification and to serum bicarbonate. Significance of the differences in the distribution was evaluated through Fisher’s test, and statistical significance was set for *p* < 0.05.

Group/Stage		IRIS 2 *n* (%)	IRIS 3 *n* (%)	IRIS 4 *n* (%)	IRIS 5 *n* (%)	*p*-Value
AKI	Total (*n* = 83)	25	20	17	21	
	No acidosis (*n* = 35)	13 (52)	13 (65)	4 (24)	5 (24)	0.0145
	Acidosis (*n* = 48)	12 (25)	7 (15)	13 (27)	16 (33)	
	Moderate acidosis	8 (32)	6 (30)	8 (47)	10 (48)	0.7383
	Severe acidosis	4 (16)	1 (5)	5 (29)	6 (28)	
ACKD	Total (*n* = 116)	12	29	39	36	
	No acidosis (*n* = 46)	5 (42)	17 (59)	12 (31)	12 (33)	0.1039
	Acidosis (*n* = 70)	7 (10)	12 (18)	27 (38)	24 (34)	
	Moderate acidosis	3 (25)	8 (27)	16 (41)	10 (28)	0.4427
	Severe acidosis	4 (33)	4 (14)	11 (28)	14 (39)	
CKD	Total (*n* = 419)	239	113	67	-	
	No acidosis (*n* = 261)	160 (67)	82 (72)	19 (28)	-	<0.0001
	Acidosis (*n* = 158)	79 (50)	31 (20)	48 (30)	-	
	Moderate acidosis	56 (23)	20 (18)	28 (42)	-	0.3496
	Severe acidosis	23 (10)	11 (10)	20 (30)	-	

**Table 3:** Kruskal–Wallis comparison for median values of serum creatinine (mg/dL), urea (mg/dL), CaxP (mg^2^/dL^2^) and bicarbonate (mmol/L) and of weight and age among AKI, ACKD and CKD groups The *p*-value was used to assess significance in the Kruskal–Wallis test, while superscript letters denoted significance in Dunn’s multiple comparison test. A *p* < 0.05 threshold was used to define statistical significance.

Parameters	Reference Range	AKI	ACKD	CKD	*p*-Value
Creatinine	0.7–1.7	4.02 ^a^ (1.82–33.48)	6.89 ^b^ (1.77–28.69)	2.53 ^c^ (1.71–19.86)	<0.0001
Urea	20–65	180 ^a^ (32–890)	289 ^b^ (49–602)	104 ^c^ (13–616)	<0.0001
CaxP	<70	70.84 ^a^ (23.8–250)	89.11 ^a^ (22.14–204)	46.26 ^b^ (10.14–290)	<0.0001
TCO_2_	14–27	16 ^a^ (2–38)	15 ^a^ (3–36)	19 ^b^ (4–45)	<0.0001

**Table 4:** A Spearman correlation analysis was conducted to examine the correlation between serum bicarbonate (mmol/L) and creatinine (mg/dL), CaxP (mg^2^/dL^2^), total calcium (mg/dL) and phosphate (mg/dL) among the AKI, ACKD and CKD groups. Statistical significance was set for *p* < 0.05.

TCO_2_ vs.	AKI		ACKD		CKD	
	Spearman r	*p*-Value	Spearman r	*p*-Value	Spearman r	*p*-Value
Creatinine	−0.33	0.0025	−0.24	0.0084	−0.17	0.0005
CaxP	−0.29	0.0074	−0.16	0.0846	−0.16	0.0007
Ca	0.07	0.5028	0.21	0.0261	0.06	0.2527
*p*	−0.35	0.0014	−0.22	0.0162	−0.21	<0.0001

**Table 5:** Fisher’s exact test was performed to assess the differences in the frequency and severity of serum bicarbonate deficiency based on a cutoff value for the serum calcium-phosphorus product (CaxP) of 70 mg^2^/dL^2^. Statistical significance was set for *p* < 0.05.

CaxP	No Acidosis	Acidosis	*p*-Value	Moderate Acidosis	Severe Acidosis	*p*-Value
<70	271 (65%)	145 (35%)	<0.0001	96 (66%)	49 (34%)	0.2147
≥70	77 (59%)	131 (65%)		77 (59%)	54 (41%)	

## Data Availability

The data presented in this study are available on request from the corresponding author due to privacy reasons.
